# Case Report: Synchronous primary high-grade appendiceal mucinous neoplasm and ovarian borderline mucinous cystadenoma: a rare case and diagnostic challenges

**DOI:** 10.3389/fonc.2026.1700388

**Published:** 2026-03-02

**Authors:** Long Chen, Yingyu Liu

**Affiliations:** Department of General Surgery, Liaoning Fangda General Hospital, Shenyang, China

**Keywords:** appendiceal mucinous neoplasm, high-grade appendiceal mucinous neoplasm (HAMN), mucinous cystadenoma, ovarian borderline tumor, synchronous tumors

## Abstract

**Background:**

Appendiceal mucinous neoplasms (AMNs), particularly high-grade (HAMN) variants, are rare and pose significant diagnostic challenges. Their presentation can mimic other abdominal pathologies, and the occurrence of synchronous primary mucinous tumors of the appendix and ovary is an exceptionally rare phenomenon that is poorly described in the literature.

**Case presentation:**

We report a case of a 59-year-old woman who presented with right lower quadrant pain and a palpable mass. Preoperative imaging identified a large, complex cystic mass suspicious for an ovarian primary. Tumor markers (CA19-9, CA125, CA15-3) were elevated. Colonoscopy revealed mucous discharge from the appendiceal orifice, shifting diagnostic suspicion. Exploratory laparotomy revealed a large appendiceal mass with cellular peritoneal mucinous deposits and involvement of the right adnexa. The patient underwent right hemicolectomy and bilateral salpingo-oophorectomy. Histopathological and immunohistochemical analysis confirmed a synchronous primary HAMN (CK20+, Villin+, CK7-) of the appendix and a primary borderline mucinous cystadenoma (CK7+, PAX-8+, CK20-) of the left ovary.

**Conclusion:**

This case underscores the diagnostic difficulty in distinguishing synchronous primary mucinous neoplasms from metastatic disease. A multidisciplinary approach, meticulous histopathological examination, and adjunctive immunohistochemistry are critical for accurate diagnosis and appropriate surgical management. This rare coexistence suggests the possibility of shared oncogenic pathways, warranting further investigation. Long-term surveillance is essential for these patients.

## Introduction

Appendiceal mucinous neoplasms (AMNs) represent a rare tumor entity, with an estimate incidence of about 1% (1, 2). They are characterized by dysplastic mucinous epithelium extending into the appendiceal wall, but lacking invasive growth or destructive invasion. Based on the degree of mucosal involvement, these tumors are classified into: Low-grade appendiceal mucinous neoplasms (LAMN), represented by mucinous cystadenoma or appendiceal borderline tumors - well-differentiated lesions with indolent growth patterns resembling adenomas. The appendiceal wall typically shows fibrosis and may contain calcifications as signs of chronic pathology. High-grade appendiceal mucinous neoplasms (HAMN), represented by mucinous cystadenocarcinoma - these tumors invade beyond the muscularis mucosae and demonstrate destructive growth patterns (3). HAMN carries a significantly higher risk of recurrence compared to LAMN. Consequently, the American Joint Committee on Cancer (AJCC) recommends staging HAMN using the same protocol as invasive appendiceal adenocarcinoma (4).

Patients with AMNs typically present with nonspecific clinical symptoms. These lesions are most frequently identified incidentally by imaging, with a subset of cases diagnosed intraoperatively or confirmed through postoperative pathological examination (1, 5, 6). Accurate preoperative diagnosis is critical, as it directly determines the surgical approach and minimizes intraoperative contingencies.

We present a rare case of synchronous mucinous tumors involving both the ovary and appendix. Preoperative evaluation of the massive abdominal mass raised suspicion of right adnexal origin, which was subsequently ruled out by colonoscopy. Postoperative histopathological examination confirmed the diagnosis of HAMN and borderline mucinous cystadenoma of the left ovary. This case report aims to enhance the clinical diagnostic rate of appendiceal mucinous neoplasms and promote standardized treatment protocols.

## Case report

The reporting of this study conforms to CARE guidelines (7). Written informed consent was obtained from the patient. A 59-year-old female patient complained of pain in the right lower quadrant, accompanied by difficulty in eating and defecation, and poor physical strength, that symptoms began one month ago. The patient is known to have high blood pressure and Type II diabetes treated with specific medication. A mass about 15cm in size could be palpated in the lower right abdomen, with poor mobility and tenderness and negative Blumberg sign.

Laboratory tests reveal the presence of elevated leukocyte count (11.11×10^9/L, normal range 3.50-9.50×10^9/L) and moderate anemia (81g/L). The tumor markers of the digestive system, CA19-9 (51.70U/ml, normal<27) and CA72-4 (10.70U/ml, normal<6.9), are elevated, while CEA and AFP are normal. The tumor markers of the ovarian system, CA15-3 (27.30U/ml, normal<25), CA12-5 (50.80U/ml, normal<35) and HE4 (176pmol/L, normal<74.3) are elevated, while β-HCG and SCC are normal. Postmenopausal ROMA is 54.1% (normal< 29.9%).

**Figure 1:**
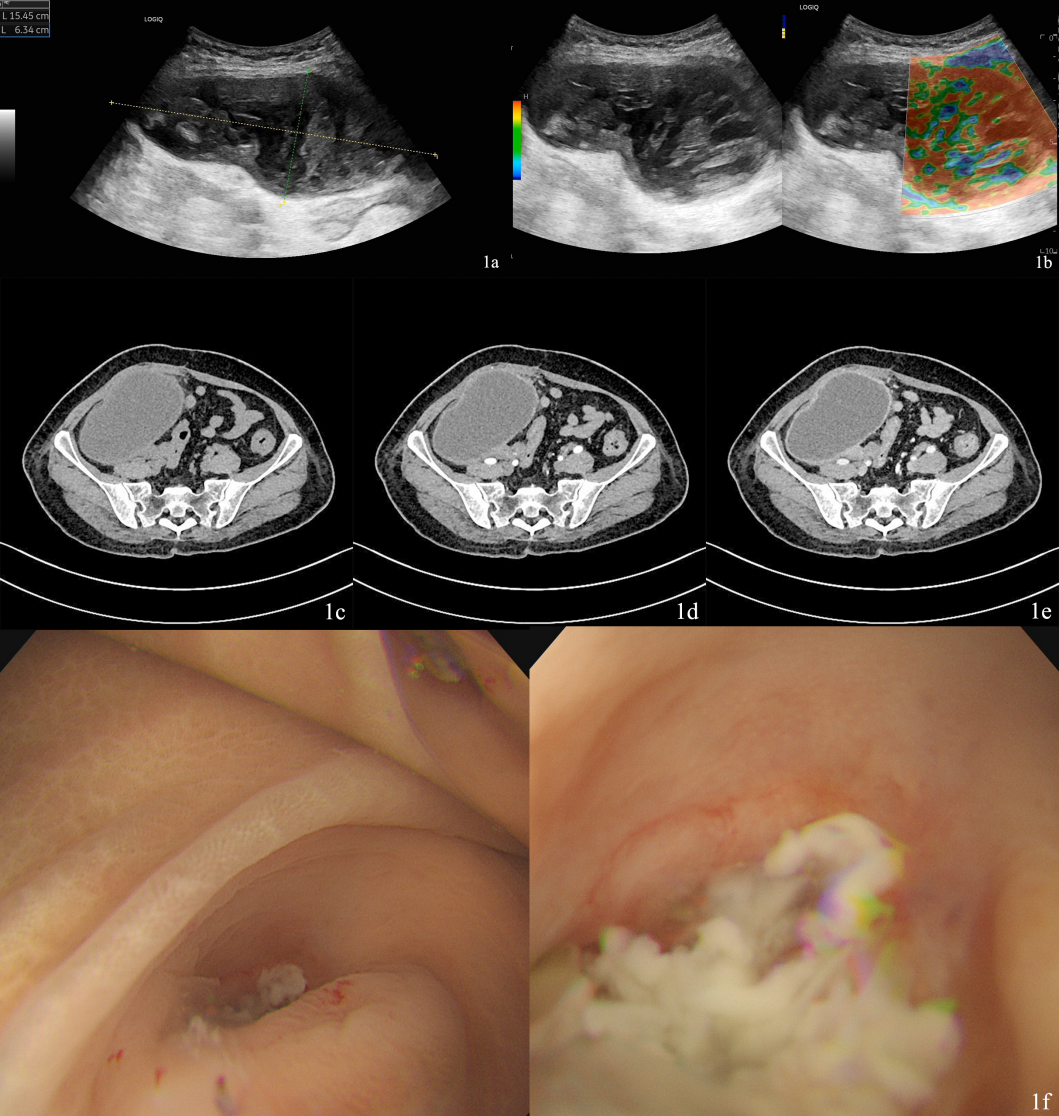
**(A)** Ultrasound showed a cystic and solid mixed mass approximately 15.4×6.3cm in size. **(B)** No intratumoral. Blood flow signals were detected. **(C)** The mass exhibits a well-defined capsule and hypodense internal contents. **(D)** Contrast-enhanced CT showed marked wall enhancement without internal vascularity. **(E)** Contrast. Enhanced CT showed marked wall enhancement without internal vascularity. **(F)** Colonoscopy showed white mucous discharge was observed at the appendiceal orifice.

Abdominal ultrasound showed a cystic and solid mixed mass approximately 15.4×6.3cm in size was observed in the right lower abdomen. The mass had a clear boundary, regular shape, and a complete capsule. It extended into the pelvic cavity and was relatively well demarcated from uterus. No obvious blood flow signals were detected within the mass (**Figures 1A, B**).

A contrast-enhanced CT of the abdomen showed a cystic hypodense mass with well-defined margins is observed in the right lower abdomen-pelvic cavity, measuring approximately 12.0 cm×7.1 cm in its largest cross-section. The lesion is predominantly cystic, demonstrating no enhancement of its cystic components on contrast-enhanced scans, while the wall exhibits marked enhancement. The mass shows ill-defined borders with adjacent segments of the right colon (cecum/ileocecal region) and portions of the small bowel. The right adnexal region displays slightly heterogeneous density and appears closely associated with the mass (**Figures 1C–E**). However, a pivotal diagnostic shift occurred with colonoscopy, which revealed mucous discharge from the appendiceal orifice (**Figure 1F**). This finding raised the first strong suspicion of an appendiceal origin for the mucinous process. Notably, preoperative imaging did not identify a separate mass in the left ovary. Evaluation for distant metastatic disease was conducted via contrast-enhanced CT of the abdomen and chest CT, which showed no evidence of hepatic, retroperitoneal, or other distant metastases. Based on the findings above, the preoperative diagnosis for this case is a mucinous neoplasm, likely originating from the appendix, with the right ovarian involvement suspected to be a secondary lesion.

Preoperative evaluation suggested infection, and antibiotic therapy was initiated. The patient’s comorbidities (hypertension and diabetes) were well-controlled preoperatively, with no contraindications to surgery. After completing preoperative preparation, the patient underwent surgical intervention. To prevent intraoperative ureteral injury, a right ureteral stent was placed prior to tumor resection. Due to the tumor size (>15 cm) and extensive adhesions, open laparotomy was preferred to minimize rupture risk.

**Figure 2:**
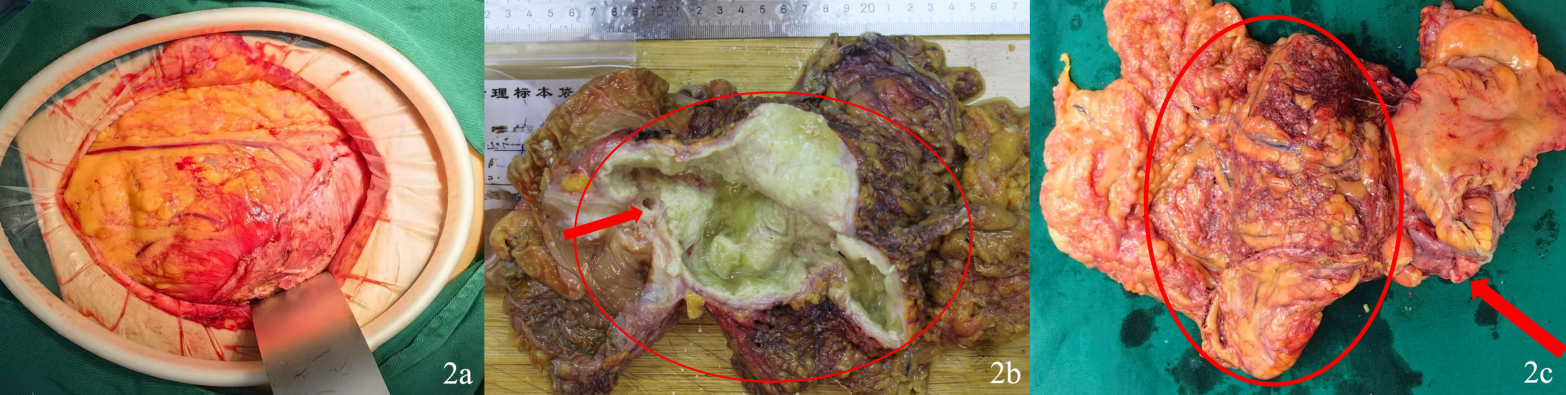
**(A)** Intraoperative photograph: A large mass was visible upon entering the abdominal cavity. **(B)** Gross photograph of the resected specimen. The appendix (the red circle highlights) is markedly dilated, measuring 15 cm in greatest dimension, and forms a large, cystic mass. Upon sectioning, the lumen is filled with abundant, tenacious, glistening mucin. The wall is markedly thickened and fibrotic. No obvious solid nodules or areas of hemorrhage are identified. The red arrow indicates the ileocecal valve. **(C)** The large tumor in the appendix is indicated by the red circle, while the right ovary is indicated by the red arrow.

Exploratory laparotomy revealed a large mass (approximately 12.0×10.0×5.0 cm) in the right lower abdomen, with significant adhesions to the colon, right adnexa, uterus, and right abdominal wall (**Figure 2A**). Mucinous deposits were observed diffusely within the peritoneal cavity. Given the findings suggestive of appendiceal mucinous neoplasm, a right hemicolectomy with ileo-transverse side-to-side anastomosis was performed. Due to dense adhesions between the right adnexa and the tumor, the right adnexa was resected en bloc with the mass (**Figures 2B, C**). Intraoperative exploration identified mucinous coating on the left ovary, prompting left salpingo-oophorectomy.

Postoperative antibiotic therapy was continued. The patient resumed oral intake on postoperative day 5, and the right ureteral stent was removed on day 14.

**Figure 3:**
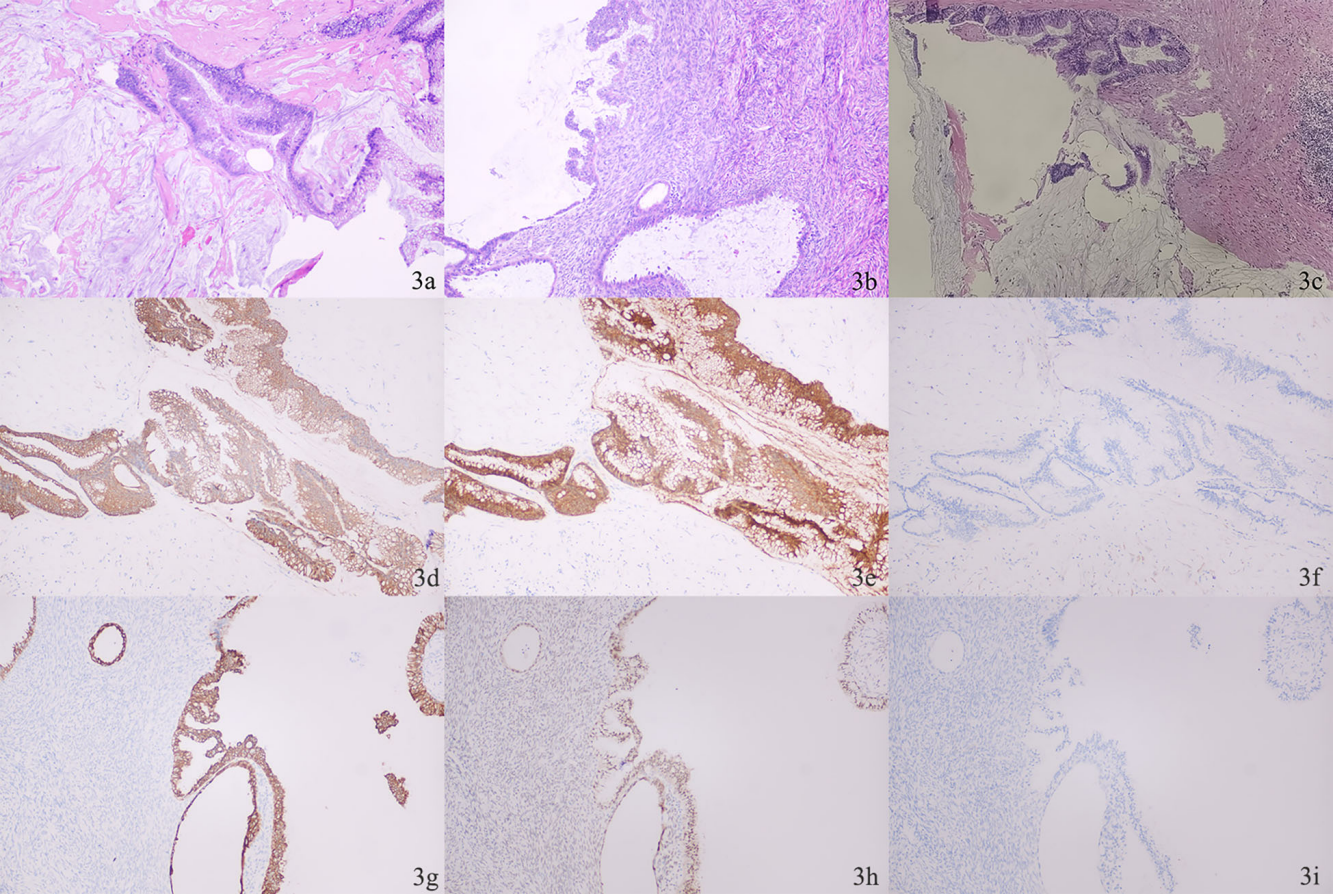
**(A)** Photomicrograph of the appendiceal neoplasm. **(B)** Photomicrograph of left ovary **(C)** Photomicrograph of mucin deposits confirmed to be cellular. **(D-F)** Appendiceal high-grade mucinous neoplasm (HAMN). **(D)** positive for CK20; **(E)** positive for Villin; **(F)** negative for CK7. **(G-I)** Left ovarian borderline mucinous cystadenoma. **(G)** positive for CK7; **(H)** positive for PAX-8; **(I)** negative for CK20.

Histopathology confirmed HAMN with en bloc invasion of the right colon and ipsilateral ovary (**Figures 3A, B**). Critically, the peritoneal mucinous deposits were confirmed to be cellular, containing strips and clusters of atypical mucinous epithelium, consistent with disseminated disease (**Figure 3C**). This diagnostic challenge was decisively resolved by postoperative histopathological and immunohistochemical analysis. The appendiceal tumor (CK20+, Villin+, CK7-, MUC4 partial+, Ki67 30%) was consistent with a primary HAMN (**Figures 3D–F**). Crucially, the left ovarian tumor demonstrated a completely different immunophenotype (CK7+, PAX-8+, CK20-) (**Figures 3G–I**), confirming a synchronous primary borderline mucinous cystadenoma and ruling out metastatic disease from the appendix. The left ovary tumor cells demonstrated ER(+), CDX-2(-), SATB2(-), CA125(+). This also explains the elevated levels of ovarian tumor markers.

These findings confirmed the appropriateness of the surgical approach, ensuring complete resection of the lesions (including the appendiceal tumor and left ovarian mass). Multidisciplinary team (MDT) evaluation determined that the patient did not require adjuvant antitumor therapy. Three-month postoperative re-examination demonstrated normalization of both CA125 and CA19–9 levels. No tumor recurrence was observed during the 6-month postoperative follow-up. Given the presentation of synchronous primary tumors, genetic counseling was recommended to assess for hereditary cancer syndromes such as Lynch syndrome. However, the patient declined germline genetic testing after counseling.

## Discussion

AMN is a rare disease caused by accumulation of secreted mucus, histopathologically classified into two distinct entities HAMN and LAMN. AMNs demonstrate a distinct female predominance, with peak incidence occurring in the 6th decade of life (2). In a landmark study, HAMN demonstrated a disease-free survival (DFS) of 21 months, with 5-year overall survival (OS) rates ranging from 45% to 65% (8). HAMN is a more rare entity that does not evolve from LAMN, with these tumors demonstrating distinct mutational profiles: HAMN predominantly harbors KRAS and GNAS mutations but rarely exhibits APC, TP53, SMAD4, or BRAF alterations, whereas LAMN more frequently involves APC, TP53, and SMAD4 mutations with infrequent GNAS mutations (9–11). The World Health Organization (WHO) provides specific criteria for diagnosis of HAMN (12).

Due to its rarity and lack of specific clinical manifestations, HAMN is frequently challenging to diagnose definitively in early stages. Differential diagnoses must include appendicitis, appendiceal perforation, and right lower quadrant masses (13, 14). The synchronous occurrence of a primary high-grade appendiceal mucinous neoplasm (HAMN) and a primary ovarian borderline mucinous cystadenoma, as confirmed in this case, represents an exceptionally rare clinical scenario. While tumor makers cannot confirm the diagnosis of AMN, they serve as a useful tool for surveillance (15). CEA, CA19–9 and CA125 assists in differentiating AMNs from other malignancies preoperatively (16). The patient demonstrated elevated CA19-9 (51.7 U/mL) and CA125 (50.8 U/mL), while CEA remained within normal range ng/mL. This pattern initially suggested primary ovarian pathology, creating diagnostic ambiguity that delayed recognition of the appendiceal origin. Besides, in some AMN cases, CA15–3 levels are elevated, which is consistent with the findings in this patient (2).

Imaging plays an important role in diagnosis. The imaging characteristics of AMNs fundamentally reflect their mucin-secreting nature. Lamellated layers of mucin demonstrate an ‘onion-skin’ appearance during ultrasonography. On MRI, intraluminal and periappendiceal mucin appears bright on T2-weighted images, while signal intensity on T1-weighted images varies depending on mucin concentration (17, 18). However, when the tumor size exceeds conventional dimensions, imaging often fails to demonstrate characteristic features (19). In this case report, the massive abdominal tumor was initially considered ovarian in origin due to its close anatomical relationship with the right ovary on contrast-enhanced CT. Preoperative diagnostic discrepancies directly dictate divergent therapeutic strategies (20). For confirmed AMNs, intraoperative caution is critical to prevent tumor rupture and subsequent peritoneal dissemination (pseudomyxoma peritonei, PMP) (21). Colonoscopy revealed white mucus at the appendiceal orifice, suggesting the neoplasm originating from the appendix. Therefore, when it is difficult to differentiate between gastrointestinal tumors and ovarian tumors, colonoscopy may provide some diagnostic assistance.

Histopathological examination remains the gold standard for definitive diagnosis of AMNs. In this case, a primary mucinous tumor was simultaneously present in the left ovary. The diagnostic challenge lay in distinguishing it from metastatic appendiceal involvement. Differentiating between primary ovarian mucinous neoplasm and ovarian metastases of appendiceal tumors depends on immunohistochemical assessment. Primary ovarian mucinous neoplasms express CK7, CK20, and PAX8 in 90%, 65-70%, and 35% of cases, respectively, while appendiceal mucinous neoplasms express CDX2 and SATB2 in 90% and 85-90% of cases (22–25). Researchers recommended the analysis of expression of at least two independent immunohistochemical markers.

Mucinous tumors most frequently arise in the gastrointestinal tract and ovaries, with frequent reciprocal metastatic spread between these sites (26–28). However, synchronous primary HAMN and ovarian mucinous tumors have not been previously reported in the literature. The distinct immunohistochemical profiles confirmed synchronous primaries, a phenomenon rarely reported. Unlike previous reports where AMN mimicked ovarian tumors radiologically (27, 28), our case is distinguished by the confirmation of two synchronous primary tumors (HAMN and ovarian borderline mucinous cystadenoma) with divergent immunohistochemical profiles, a scenario not previously documented in the literature. Notably, imaging failed to identify the appendiceal origin due to the tumor’s massive size and adhesion to the ovary, underscoring the necessity of colonoscopy and IHC in ambiguous cases. This case underscores the importance of multidisciplinary team (MDT) collaboration in managing synchronous tumors. En bloc resection may be preferable to avoid incomplete excision. The identification of cellular mucin in our case further reinforced the need for radical surgery and meticulous peritoneal inspection. Given the rarity of such cases, long-term surveillance with CA125/CA19–9 and imaging is advised to detect recurrence early. Particularly in the setting of cellular peritoneal dissemination, close monitoring is paramount. The application of adjuvant chemotherapy in AMNs is controversial and has not been evaluated in a prospective study.

Although HAMN and ovarian mucinous tumors arise independently, their shared mucin-secreting phenotype raises questions about potential molecular links. Both tumor types are known to harbor KRAS mutations (9, 22), suggesting a possible common oncogenic driver, although the absence of GNAS mutation in the ovarian tumor in our case argues against direct clonal relatedness. An alternative hypothesis is a peritoneal “field effect” that predisposes to independent mucinous neoplasia. Furthermore, the presentation warrants consideration of an underlying hereditary predisposition, such as Lynch syndrome, which increases the risk for various carcinomas including those of the ovary (29). However, definitive insights are limited in our report. The postoperative follow-up period remains short, and molecular profiling of the tumors was not performed, precluding confirmation of shared mutations or mismatch repair deficiency. Additionally, while genetic counseling was recommended, germline testing was declined by the patient.

## Conclusion

Accurate diagnosis of AMNs remains challenging, particularly when tumors exceed conventional size limits and exhibit ill-defined ovarian borders, increasing the risk of misdiagnosis. To ensure optimal surgical decision-making and favorable prognosis, comprehensive preoperative evaluation is essential for definitive diagnosis. Crucially, both colonoscopy and immunohistochemical analysis played indispensable roles in achieving the correct diagnosis. MDT discussions hold significant value in clinical management. Furthermore, the presentation of synchronous primary tumors warranted consideration of an underlying hereditary cancer syndrome, although definitive genetic assessment was not performed per patient preference. The presence of synchronous tumors may suggest shared oncogenic pathways, warranting further investigation. Long-term postoperative surveillance is critical for early detection of recurrence.

## Data Availability

The raw data supporting the conclusions of this article will be made available by the authors, without undue reservation.
